# Multimodal brain imaging with magnetoencephalography: A method for measuring blood pressure and cardiorespiratory oscillations

**DOI:** 10.1038/s41598-017-00293-7

**Published:** 2017-03-14

**Authors:** Teemu Myllylä, Norman Zacharias, Vesa Korhonen, Aleksandra Zienkiewicz, Hermann Hinrichs, Vesa Kiviniemi, Martin Walter

**Affiliations:** 1 University of Oulu, Optoelectronics and Measurement Techniques Research Unit, Health & Wellness Measurements Group, Oulu, Finland; 2 Leibniz Institute for Neurobiology, Magdeburg, Germany; 3 Charité – Universitätsmedizin Berlin, Department of Anesthesiology, Neuroimaging Research Group, Berlin, Germany; 4 Oulu University Hospital, Department of Diagnostic Radiology, Oulu, Finland; 5 University of Oulu, Research Unit of Medical Imaging, Physics and Technology, Oulu Functional NeuroImaging Group, Oulu, Finland; 6 University Hospital Magdeburg, Clinic for Neurology, Magdeburg, Germany; 7 University of Tübingen, Department of Psychiatry, Tübingen, Germany

## Abstract

Studies with magnetoencephalography (MEG) are still quite rarely combined simultaneously with methods that can provide a metabolic dimension to MEG investigations. In addition, continuous blood pressure measurements which comply with MEG compatibility requirements are lacking. For instance, by combining methods reflecting neurovascular status one could obtain more information on low frequency fluctuations that have recently gained increasing interest as a mediator of functional connectivity within brain networks. This paper presents a multimodal brain imaging setup, capable to non-invasively and continuously measure cerebral hemodynamic, cardiorespiratory and blood pressure oscillations simultaneously with MEG. In the setup, all methods apart from MEG rely on the use of fibre optics. In particular, we present a method for measuring of blood pressure and cardiorespiratory oscillations continuously with MEG. The potential of this type of multimodal setup for brain research is demonstrated by our preliminary studies on human, showing effects of mild hypercapnia, gathered simultaneously with the presented modalities.

## Introduction

Combining different brain imaging techniques enables us to study the causality between complex neurological mechanisms and variables. For instance, an increase in neuronal activity causes a metabolic demand for glucose and oxygen, which increases cerebral blood flow to the active brain region. This kind of process is impossible to study accurately by any single imaging modality, but requires simultaneous use of hemodynamic and electromagnetic based imaging techniques. In consequence, multimodal imaging, such as electroencephalogram (EEG) with magnetoencephalography (MEG) or functional magnetic resonance imaging (fMRI) is a common practice in modern day neuroimaging. In addition, such imaging techniques would draw an advantage if cardiovascular and cerebral hemodynamic related signals are recorded comprehensive and in synchrony. This would extend our possibilities to acquire detailed knowledge of the functional interconnections between the brain and other organs and, for example, to study autoregulation of blood pressure (BP).

MEG, closely related to EEG, measures the magnetic fields created by the electric currents, whereas EEG measures electric potentials by electrodes placed at certain points on the scalp. The main difference between the sources of EEG and MEG signals is that MEG only picks signals from the dendrites tangential to the head surface. Both of these methods can directly measure neuronal activity with a time resolution of less than one millisecond and with a high amount of channels, commonly MEG from 100 to 300^1^. In general, EEG has a relatively modest spatial resolution, on the centimetre scale, whereas MEG has a higher spatial accuracy, few millimetres under favorable conditions^2, 3^. Commonly, MEG has been combined with EEG, for example in study of epilepsy, because MEG and EEG complement each other for the detection of interictal epileptiform discharges (IED)^4^. More recently, avalanches of neuronal activity were studied by using magneto-electroencephalography (MEEG)^5^. The successive individual neuronal avalanches arriving into brain areas fuse into a fluctuating signals that extend through the neuronal networks forming continuous streams of information. Recent ultra-fast magnetic resonance imaging sequence called magnetic resonance encephalography (MREG) has been able to capture spreading of highly similar avalanches following network topology deeper into the brain tissue^6^.

Since MREG enables critical image sampling, it enables separation of cardiorespiratory pulsations and very low frequency (VLF) vasomotor waves from electrophysiological avalanches. The physiological pulsations have become increasingly important as they are the drivers of glymphatic brain clearance mechanisms^7^. The VLF waves are being reflected in combined direct current (DC)-EEG - near-infrared spectroscopy (NIRS) measurements which reflect the stability of the neurovascular unit^8, 9^. However the magneto-electric properties of the VLF vasomotor waves are still not very well understood/researched.

While combination of blood oxygenation level dependent (BOLD) changes and electrophysiology can easily be done for EEG via fMRI measurements, this is not possible if MEG specific information is sought for. Furthermore, for crossmodal correspondence in high temporal resolution combination of fMRI and EEG is not suitable. Combination of EEG and fNIRS may provide necessary temporal resolution but loses spatial accuracy. In multimodal brain studies it is desirable that at least one modality provides adequate spatial information. Thus, to realize this issue, MEG can incorporate EEG measures but relies on optical BOLD assessments. While fNIRS and BP provide important information for hemodynamic forward models, their applications in MEG measurements are equally desirable.

As MEG has good spatial resolution combined with excellent temporal resolution, we set out to combine multimodal MEG setup enabling, probably for the first time, simultaneous use of MEG with continuous measurements of cerebral hemodynamic, cardiorespiratory and BP oscillations. Developed method to quantify cerebral hemodynamics is based on NIRS^10^ and the method to continuously estimate BP oscillations is based on determining pulse propagation between heart and carotid artery, assumed to reflect systemic BP^11^. Further, heart rate (HR) and breathing patterns are determined by measuring chest wall motion. In the following, we introduce our multimodal MEG setup utilizing fibre optics. In particular, the method for measuring BP oscillations is presented. In results section, the MEG compatibility of the exploited measurement methods is verified. Further, we demonstrate that pulse transit time (PTT), for estimating BP oscillations, is possible to determine in MEG, at simplest, by using only one fibre optics based sensor. Lastly, we present effects of mild hypercapnia induced by breath holding in the gathered signal modalities. Due to lack of statistical evaluation these are shortly discussed, but aim to demonstrate the potential of this type of multimodal setup for brain research.

### The advantage of fibre optics for use in MEG environments

**Figure 1:**
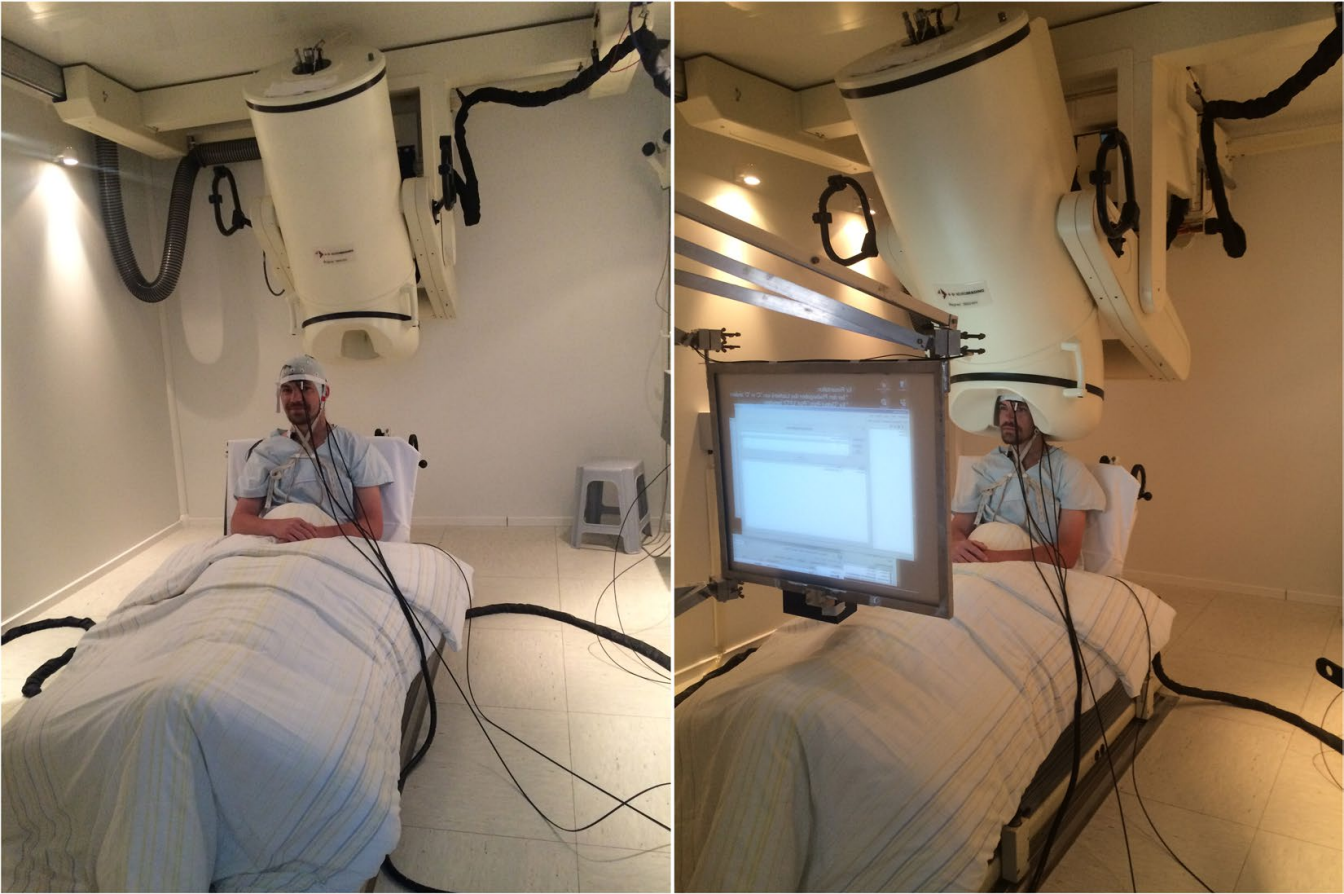
Multimodal setup in MEG environment. In the pictures, the black cables with fibre optics based sensors attached on the body and head are optical fibres for measurements of cNIBP and NIRS. On the right, shown is performing of simultaneous recording with the setup. During imaging, the display is used to instruct the subject to perform the given task.

MEG device is placed inside an electromagnetically shielded room to enable sufficient signal to noise sensitivity for weak magnetic field variations, in range of 1 fT to 1 pT, near the surface of the head. Hence, even very low level electromagnetic fields inside the MEG chamber can disturb the measurement which highly restricts the use of additional measurement devices inside the chamber. Moreover, materials used in instruments and sensors inside the MEG helmet must be non-magnetic. Consequently, use of optical fibres combined with MEG is desirable, since they can be fully made of glass or plastic, which are non-magnetic and electrically non-conductive materials. Further, only light is used as a measurement signal. Fortunately, cerebral hemodynamics, BP and cardiorespiratory signals are possible to measure utilising methods and materials required by the MEG environment. In the presented setup, all electrical parts of the equipment are placed outside the MEG chamber and only the sensors and optical fibres were brought inside the MEG chamber. The accelerometers (ACM), used for sensing cardiovascular and respiratory signals, rest on using opto-mechanical techniques, presented in Myllylä *et al*.^11^ and measurement of cerebral hemodynamics employs NIRS technique presented in Sorvoja *et al*.^12^. Figure 1 shows the MEG environment utilizing fibre optics for measuring cerebral hemodynamics, BP and cardiorespiratory signals simultaneously with MEG.

In the setup, BP oscillation is estimated based on the mutual relationship between pulse wave velocity (PWV) and BP; in general, the higher the pressure in the aorta is, the greater is the velocity of blood flow. The method is discussed for example in papers by Yoon *et al*.^13^ and McCarthy *et al*.^14^. PWV can be derived from PTT which is commonly determined between the ECG R peak and the corresponding pulse wave detected by PPG sensor, which is normally placed on the fingertip.

**Figure 2:**
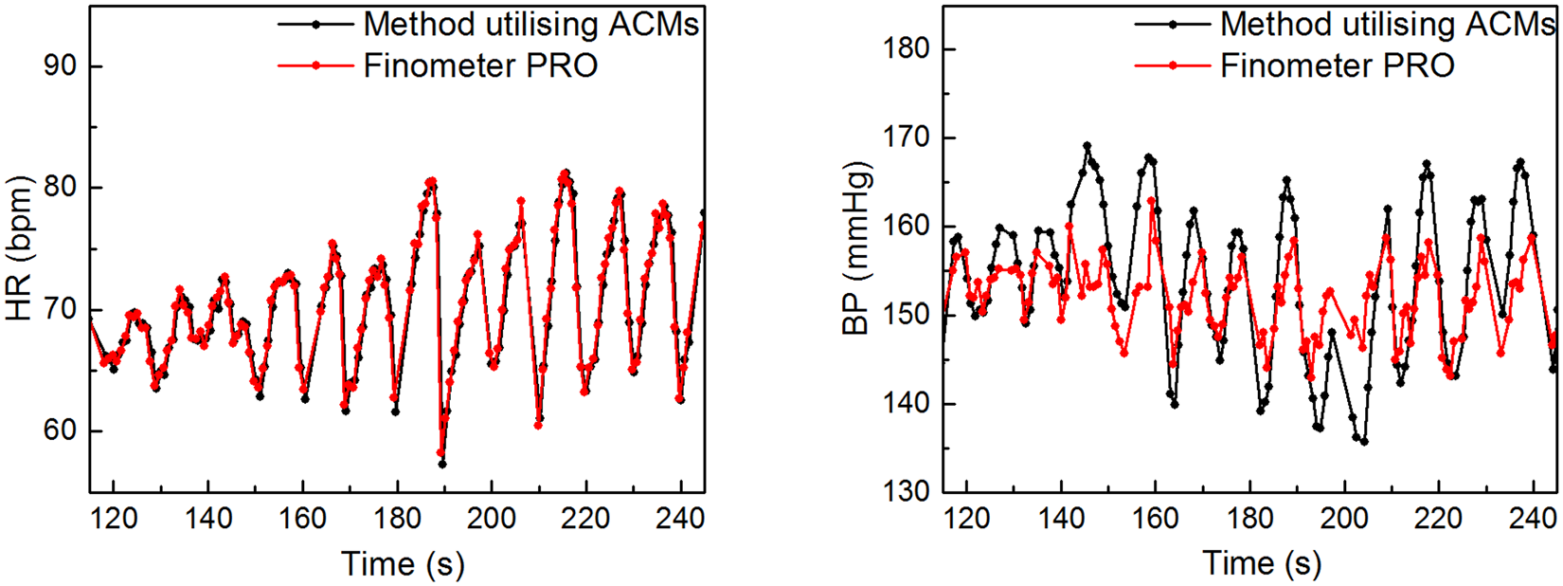
A comparison of signals for HR (on the left) and BP (on the right) when measured simultaneously with Finapres and with the developed MEG compatible cNIBP device, based on PTT measured with optical ACMs. The correlation for BP signals is r > 0.7 and for HRs r > 0.95. The BP estimation from the PTT is based on the formula presented in publication by Gesche *et al*.^27^.

PTT can be determined also by using ACMs^15^. We have previously developed an MRI compatible continuous non-invasive BP (cNIBP) measurement method^11^ which was now utilised for the first time in MEG environment. In the method, one ACM is placed on neck and accurately senses skin vibrations which reflect the pulsation of carotid artery in the proximity of the ACM. Another ACM is placed on chest and it is sensitive to chest wall movements caused by heart beat and breathing. For the PTT calculation, the ACM on chest gives corresponding signal for ECG R peak, which is convenient because ECG is often too disturbed when measured during MRI. The method has been found advantageous in use with multimodal MRI studies^9^ and its accuracy to estimate BP looks promising, as demonstrated in Figure 2 (on the right), showing the response concurrently with the Finometer®PRO (Finapres Medical Systems), which is widely used in clinical settings for non-invasive continuous BP measurements. Further, as Figure 2 (on the left) shows, also HR can be accurately determined using the ACMs. The precise validation of the BP accuracy of the cNIBP is still in process and, for instance, improvements in signal processing for reducing motion artefacts are still required. On the other hand, motion artefacts are less present when using the method particularly in MEG environment.

**Figure 3:**
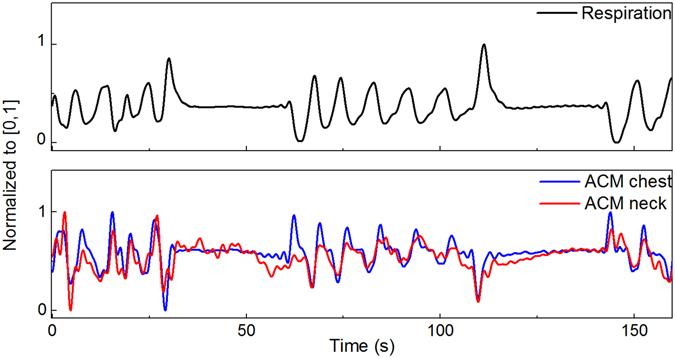
Breathing signal measured by respiration belt (top) and ACMs (down) during breath hold task. As can be seen breathing signal can be easily tracked also with ACMs placed on chest and neck. ACM placed on chest is more sensitive and corresponds better to respiration belt. In the ACM signal, corresponding breathing signal was processed by using a 0.5 Hz low pass filter^16^. Breath hold task was carried out between 30–60 s and 110–140 s.

In addition, the ACMs can be used for detecting breathing patterns, especially when subject is sitting or lying^16^. Figure 3 shows breathing patterns gathered with the ACMs on chest and neck concurrently with the breathing response measured by an anaesthesia monitoring device when using a belt during a breath hold task. In MEG studies, breathing is considered commonly as an artefact generator, because subject’s breathing causes slight movements of the head. This may lead to a shift of the brain regarding the fixed sensor of the MEG-dewar (the part of the MEG system where the head is put in) that might be visible as a shift of energy of neighbouring sensors which shifts back when the subject breath out. Such shifts should be avoided and an additional breathing signal could be potentially utilised for instance to regress these kind of artefacts.

## Results

**Figure 4:**
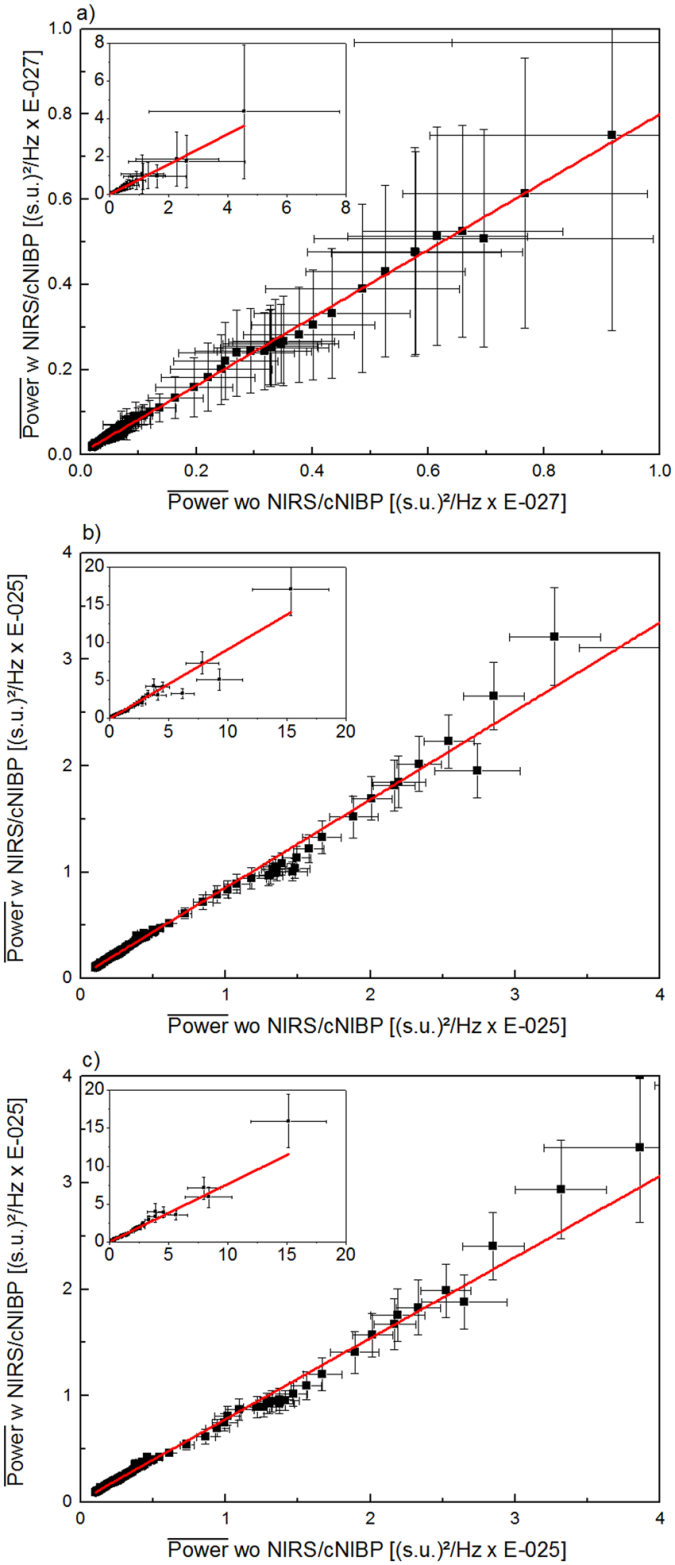
Comparison of mean power values (black dots, whiskers = SD) measured with magnetometers (**a**) and planar gradiometers (**b** and **c**), with (x axis) and without (y axis) the multimodal setup. For better visualisation the plots show an extract of all data, which were shown in each inset separately. In all cases the data can be well explained by a linear fit (red line). Note the slight positive intercept of the linear fits due to smaller power values when the multimodal setup is applied. The external sensors for NIRS are attached on back of the head and on forehead above left eye. Sensors for cNIBP are attached on chest and neck (see Figure 1).

Possible interferences in the MEG data were observed to verify MEG-compatibility of the utilized cNIBP and NIRS devices for different MEG sensor types (Magnetometer and Gradiometer). When comparing the measured signal with and without the NIRS and cNIBP devices we observed quasi no influence on the quality of the measurement. In Figure 4 we plotted the resting state mean power values averaged over all channels plus standard deviation for condition with cNIBP and NIRS devices attached versus condition without cNIBP and NIRS devices attached. Linear fits of the data show very good correlations with high Pearson R^2^ > 0.99 (Figure 4a: magnetometer Pearson R^2^ = 0.993, 4b gradiometer coil 1 Pearson R^2 ^ = 0.993, 4c gradiometer coil 2 Pearson R^2^ = 0.991). In all cases a positive intercept of the linear fit was detectable with values smaller than 1% and 2% of the mean power values for magnetometer data and gradiometer data respectively (4a: 0.004E-27 T^2^/Hz ± 1.985E-31 T^2^/Hz, 4b: 0.016E-25 T^2^/Hz ± 9.493E-29 T^2^/Hz, 4b: 0.019E-25 T^2^/Hz ± 0.001E-29 T^2^/Hz). For original data see supplementary material.

### BP estimation utilising heart beats detected with MEG

**Figure 5:**
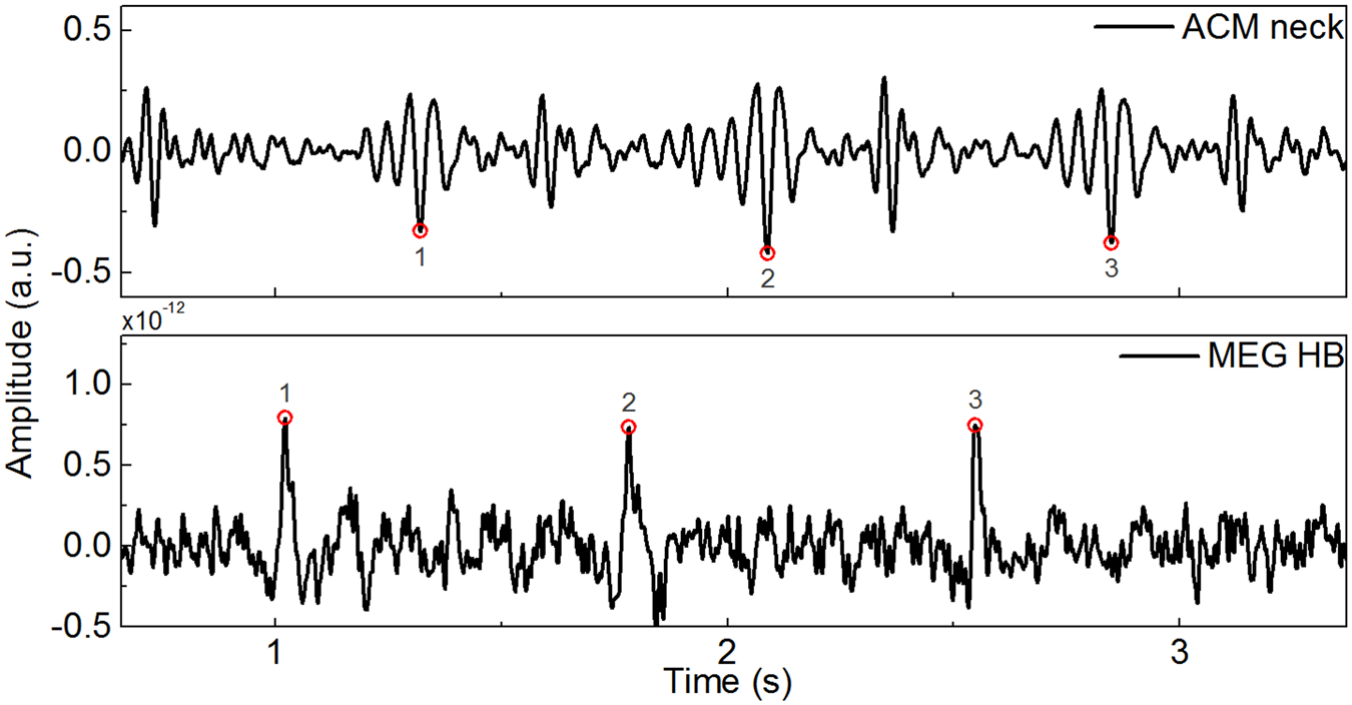
Raw signals recorded during resting state. Lower signal showing peaks for heart beats is recorded by one MEG channel and above it is shown response of the ACM placed on the neck of the patient. In both signals the heart pulsation can be observed.

**Figure 6:**
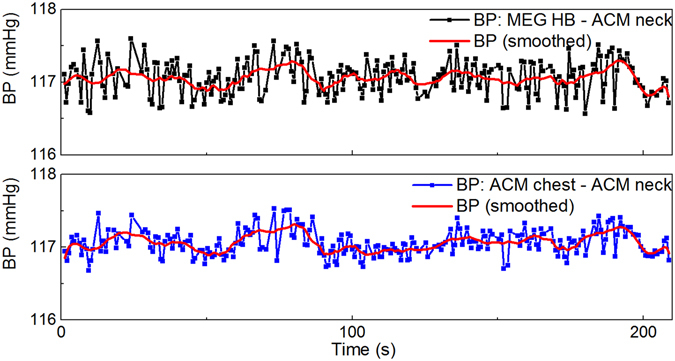
A comparison of BP oscillations when using two PTT calculation methods simultaneously. In the upper plot, BP is based on the PTT calculation between when ACMs are placed on chest and neck. In the lower plot, PTT is between heart beats determined by MEG and ACM placed on neck. The values calculated for BP are not absolute, because the calibration procedure was omitted. Red curve shows averaged responses for PTT.

MEG is partly sensitive to electrical activity of heart. In MEG studies, these are normally considered as artefacts^17^. We studied the possibility to use MEG data of separate channels to calculate PTT, between heart beats recorded by MEG and ACM placed on neck. This would enable us to estimate BP oscillations during MEG by using only a single ACM (alternatively, also MEG compatible PPG sensor could be used). Figure 5 shows an example of a raw data including heart beats measured by one MEG channel and response of ACM when placed on neck. MEG signal clearly shows higher peaks, representing heart beats, which correspond to the delayed beats visible in ACM signal. Further, Figure 6 shows two estimations for BP oscillation based on the PTT method. The measurement was performed in MEG using a resting state task. In the upper plot, one ACM was placed on chest and the other on neck while the lower plot represents BP estimation using PTT calculation between heart beats of a MEG channel and one ACM, placed on neck. As it can be seen, both methods give similar fluctuation for continuous BP signals, with the correlation of approximately 0.8.

### Effects of mild hypercapnia recorded simultaneously with the presented modalities

**Figure 7:**
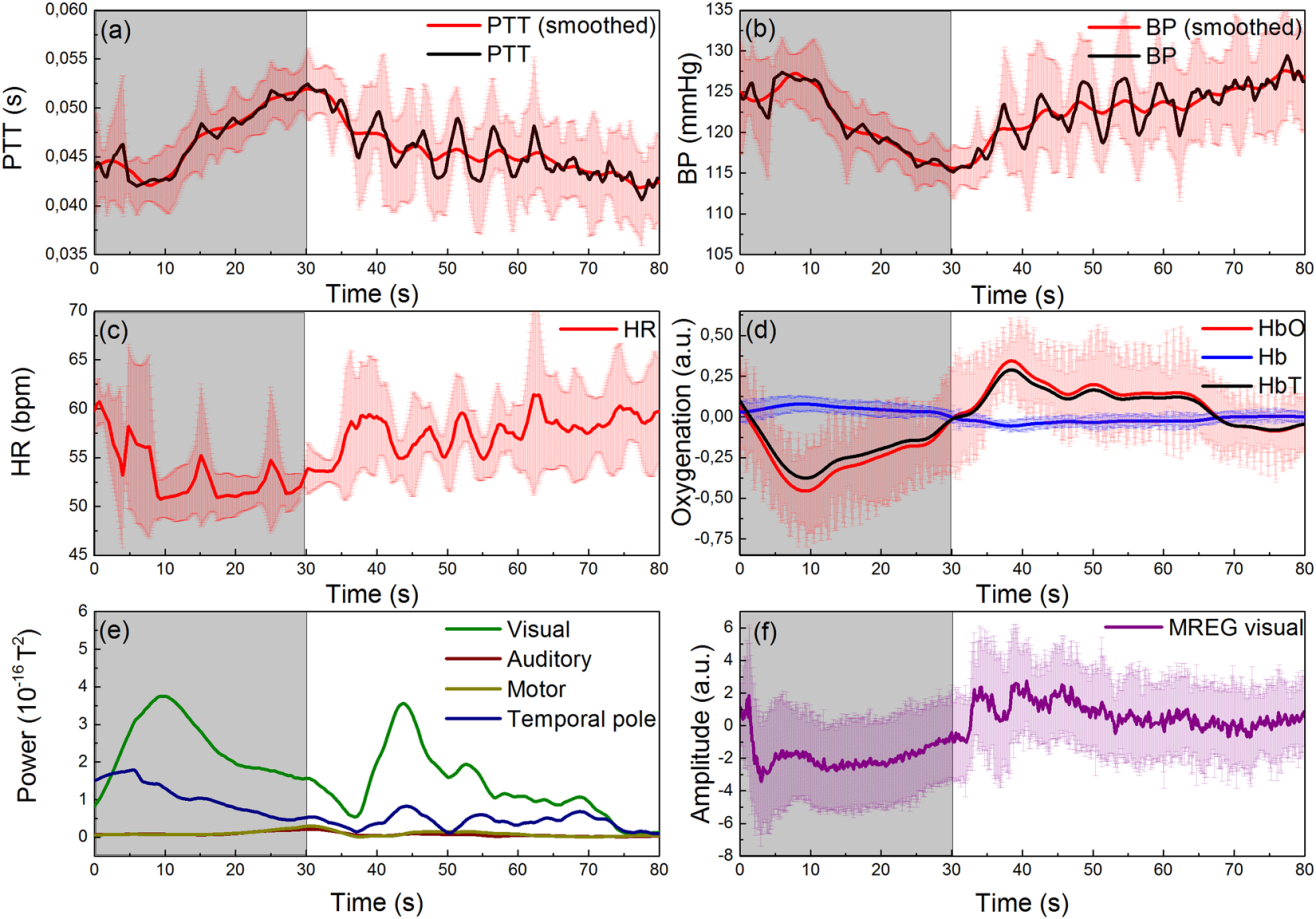
Averaged responses for PTT (**a**), BP (**b**), HR (**c**) measured with the cNIBP device; HbO/Hb (**d**) measured with the NIRS device from back of the head and four scouts (MEG) covering visual (green line), auditory (brown line), motor (dark yellow line) and temporal pole (blue line) brain areas (**e**). Average for NIRS and cNIBP data was calculated using values obtained during 4 breath holding sequences. Vertical bars represent standard deviation of the values. In addition, (**f**) shows an averaged response of 8 breath holds using the same breath hold task but recorded independently with our MREG multimodal setup^9^. In all subfigures t = 0 marks the beginning of breath holding for 30 s, highlighted with grey area.

In addition to technical validation of the setup, we performed simultaneous multimodal measurements altogether for 4 volunteer subjects, focusing on effects of mild hypercapnia. In the mild hypercapnia experiment the subjects were instructed to sit quietly in the MEG chamber, to take a deep breath when a marker appears on a screen and hold on for 30 s until the marker disappears. After this so called breath hold manoeuvre the subject had time for normal breathing. After each breath hold a second marker appears for 30 s where the subject had to breathe normal. The whole procedure was repeated five times. Figure 7 shows averaged responses of PTT, BP, HR and HbO/Hb for breath holding condition (7a to 7d) and the source strength of four scouts covering different brain regions (e; light green = visual cortex, brown = auditory cortex, dark green = temporal lobe and yellow = motor cortex) gathered from MEG. When the 30 s long breath holding manoeuvre starts at t = 0 HbO shows a transient decrease in signal strength for this duration whereas a slight transient increase can be observed for Hb. Such change in HbO with just a slight change in Hb seems to evoke neural activation just in visual brain areas, as only the scout covering visual cortex show an increase in activation with highest deflection at t = 9.61 s (see Figure 7e). This result was verified using ultra-fast MREG BOLD signal from visual cortex in 8 subjects. BOLD signal also drops during breath hold and then overshoots after breath hold in nearly identical manner to HbO, HbT and BP data, cf. Figure 7f.

## Discussion

In our multimodal measurements, optical fibres based methods were shown to be mostly suitable to be utilized in MEG imaging environment. No interference caused by the additional devices was found in the averaged magnetic field of the MEG (Figure 4). Importantly, in the applied methods, only optical fibres and optics based sensors were used inside the MEG chamber. The NIRS sensors were attached between head surface and the surface of the MEG dewar. As a consequence an increased distance of the subjects head to the surface of the MEG dewar can be seen. But, with increased distance between head surface and sensors the signal strength will decrease with 1/r^2^. This results in smaller signal strength values when the NIRS sensors were applied and in positive intercepts of the linear fits to the data (Figure 4).

BP oscillations are commonly estimated based on the PTT method, and the PTT is usually measured between the ECG R peak and the corresponding maximum inclination in the PPG signal. Our experiments indicate that, it is possible to use the heart beat detected by MEG (Figure 6), as a corresponding ECG R peak for PTT calculation. Thus, during MEG imaging we are able to determine PTT by using a single ACM to estimate BP oscillations. All in all, by using only one MEG compatible ACM we can acquire signal to calculate HR, breathing and BP oscillations simultaneously with MEG.

Presented effects of breath hold task obviously require statistical validation since only 4 subjects participated the experiments. Though, it emphasizes the potential of this type of multimodal setup for brain research. During breath hold, HR fluctuates and slightly decreases while BP increases. This is followed by a decrease in blood oxygenation while Hb stays mainly constant (see Figure 7a–d) and a neural activation of brain regions covering visual brain areas (Figure 7e). Other brain areas like auditory cortex or motor cortex were not affected. This is in line with other publications, which examine the effects of the Valsalva maneuver^18^ or induce hypercapnia^19^ in human subjects. An effect related to breathing could be excluded by comparing the different scout activations. In all scouts the effect of breathing at the beginning of the breath hold manoeuvre is clearly visible as a loss of activation right at the beginning of the 30 s breath hold. After this dip in signal strength the activation strength of all but the occipital scouts reached comparable activation levels as before breath hold. In addition, HbO measured by NIRS from occipital cortex showed high correlation with MEG power in occipitotemporal areas (Figure 7e,d). It was recently shown that CO_2_ level increase during hypercapnia also reduces MEG power in wide range^20^. Here we further elaborate the same result and show that in addition to MEG power reduction also the BP reduced in the brain simultaneously to MEG power drop in occipitotemporal areas. We have also similar findings in ultrafast fMRI MREG sequence showing arterial pulsation amplitude drop during identical 30 sec breath hold. The effects of HbT are assumed to be mostly caused by the changes in cerebral blood flow due to fluctuations in BP and HR, when the cerebral autoregulation, after a delay, aims to maintain blood flow sufficient to the brain during changes in BP. However, the physiological mechanisms of the cerebral autoregulation is more complex^21, 22^ and the relationship between BP and cerebral hemodynamics is not always that obvious.

In this article, we show, to the best of our knowledge for the first time, that continuous measurement of BP oscillations can be performed simultaneously with MEG without relevant MEG signal degradation. In addition to cNIBP, the NIRS method was utilised to estimate BOLD and metabolism in cerebral cortex simultaneously with MEG. Presented NIRS, cNIBP and MEG rely on different sensing techniques thus cannot be directly affected by each other but shown correlations are presumed to be caused by the physiological effects due to interactions between the brain and body, particularly the cardiovascular system. These interactions can be studied only by using a multimodal setup such as presented here. Interestingly, although sill preliminary, we noticed that during breath hold all physiological changes seemed pointing in the same direction; reduction of BP, oxygenation and MEG power. However, this still requires further studies particularly focusing on statistical evaluation of the result.

## Methods

The study was performed in accordance with the guidelines approved by the ethics committee of the Medical Faculty of the Otto-von-Guericke University. Simultaneous MEG, NIRS and cNIBP measurements were performed altogether for 4 volunteer healthy subjects (1 female and 3 males with ages between 25 and 35 years). All subjects gave their informed consent prior to the start of the experiment.

### Cerebral hemodynamics

The NIRS device applied to the MEG environment uses frequency domain (FD) technique^12^. Optical fibres used in the NIRS device are 10 meters long having 4 light source inputs and one output tip to enable mixing of four narrow band wavelengths, each modulated at a specific frequency. NIR light is produced by selectable laser diodes (LD) and high-power light emitting diodes (HPLED). The fibre output tip with a 90 degree bend is attached on the head using a fibre clip made of plastic. The diameter of the tip is 2.5 mm. All fibres have the same physical dimensions.

Measured raw NIRS time courses were converted into two time courses representing temporal changes in HbO and Hb concentrations using Matlab-based NIRS data analysis tool HoMer2. HoMer2 is open source software which calculates concentration changes of Hb, HbO and HbT automatically, based on MBLL, from the selected raw NIRS data when all the needed parameters are given. The basics of the program can be found in the publication by Huppert *et al*.^23^. In the performed measurements, wavelengths of 660 nm and 850 nm were used. According to our studies, this wavelength combination gives a good sensitivity for HbO, Hb and HbT changes^10, 24^. Two NIRS optodes were used; one attached on subject’s occiput and the other on forehead, both having a source-detector distance of 3 cm. The sampling rate of the NIRS data acquisition was 1 kHz.

MREG BOLD data verification was done using an identical system to out previous multimodal MREG system during a 32 s breath hold repeated 5 times^9^. FSL MELODIC was used to identify BOLD signal source from visual cortex after standard pre-processing of the MREG data.

### MEG recording & analysis

The measurements were performed in Clinic for Neurology at the University Hospital Magdeburg, Germany, in the MEG lab equipped with a 248-channel whole-head device (BTI Magnes 3600 WH), shown in Figure 1, and a newly established 306-channel Elekta Neuromag TRIUX MEG system (Elekta-Neuromag Oy, Helsinki, Finland, not shown). The Elekta Neuromag TRIUX MEG system was used for compatibility testing, because it allows to measure the effects on different sensor types (magnetometer and gradiometer) at the same time. MEG signals were acquired with a sampling rate of 1 kHz. For compatibility testing of the cNIBP/NIRS setup, the data were acquired with an online filter of 0.03 Hz to 330 Hz. Additionally the Elekta Neuromag MaxFilter was applied offline. In all other measurements, performed with the BTI Magnes 3600 WH, an online low pass filter of 100 Hz was applied. For analysis of MEG-resting-state data a band pass filter of 0.01 Hz to 0.08 Hz and for task data a band pass filter of 0.5 Hz to 30 Hz was used. For MEG-breath holding data a time interval of 5 s pre stimulus to 80 s post stimulus of all breath holding time points was defined. After baseline correction by the use of the mean amplitude value over the whole time interval, the high-pass filtered (0.01 Hz) data intervals were averaged. For source analysis a distributed source model (sLORETA^25^) with a 3 shell spherical head model was applied. During this approach the brain surface was divided into 15002 vertices and the resulting source strengths of the vertices were used for further analysis. Here we focus on four predefined brain region of interest (Scouts) covering auditory, visual, motor and temporal pole areas of the right hemisphere with a mean vertices amount of 78.8 ± 8.5 and a mean area of 13.08 ± 0.05 cm^2^ of the cortical surface. For analysis we compute the mean amplitude and power values of these Scouts. Offline filtering, averaging and source modelling was done via Brainstorm^26^. For power analysis we performed a power spektrum density analysis using Welch's method implemented in Brainstorm.

### PTT, HR and BP analysis

Pulses for calculating the PTT and HR were identified from the ACM signal by searching for the maximum value of each time interval in synchronously measured signals, by using Matlab. In determining these time intervals, the signal response with the clearest and most obvious peak was chosen as a reference. To ensure that the right peaks were identified, the peaks were marked as dots on the signal curves and then checked visually that they are in the right places. Further, PTT was calculated using the methods presented in Myllylä *et al*.^11, 15^. The BP estimation from the PTT is based on the formula presented in publication by Gesche *et al*.^27^.
